# Anti-Apoptotic Effects of Resistance Training and Tribulus Terrestris Consumption in the Heart Tissue of Rats Exposed to Stanozolol

**DOI:** 10.5152/eurasianjmed.2021.20051

**Published:** 2021-06

**Authors:** Ayat Arjmand, Bahram Abedi, Seyed Ali Hosseini

**Affiliations:** 1 Department of Physical Education and Sport Sciences, Mahallat Branch, Islamic Azad University, Mahallat, Iran; 2 Department of Sport Physiology, Marvdasht Branch, Islamic Azad University, Marvdasht, Iran

**Keywords:** Training, *Tribulus terrestris*, Heart, Stanozolol, Apoptosis

## Abstract

**Objective:**

Nowadays, the use of energetic substances has become a complex problem in sports, such that the role of anabolic-androgenic steroids is undeniable. This study aimed to investigate the antiapoptotic effect of resistance training and *Tribulus terrestris* in the heart tissue of rats exposed to stanozolol.

**Materials and Methods:**

35 rats divided into 7 groups including (1) sham, (2) stanozolol-treated, (3) stanozolol+50 mg/kg *Tribulus terrestris*-treated, (4) stanozolol+100 mg/kg *Tribulus terrestris*-treated, (5) stanozolol+resistance training-treated, (6) stanozolol+resistance training+50 mg/kg *Tribulus terrestris*-treated, and (7) stanozolol+resistance training+100 mg/kg *Tribulus terrestris*-treated. During 8 weeks, groups 2–7 received 5 mg/kg stanozolol per day peritoneally; groups 5–7 performed resistance training for 3 sessions per week; and groups 3, 4, 6 and 7 received daily doses of *Tribulus terrestris* peritoneally.

**Results:**

Stanozolol administration significantly increased the BAX, BCL-2, P53, and caspase 3 and BAX/BCL-2 ratio (*P* < .001). Resistance training, 100 mg/kg *Tribulus terrestris* administration, 50 mg/kg Tribulus terrestris administration, resistance training+100 mg/kg *Tribulus terrestris* administration, and resistance training+50 mg/kg *Tribulus terrestris* administration significantly decreased BAX, BCL-2, P53, and caspase 3 levels and BAX/BCL-2 ratio (*P* < .001); however, stanozolol+resistance training+100 mg/kg *Tribulus terrestris* administration caused more decrease than stanozolol+resistance training+50 mg/kg *Tribulus terrestris* administration in BAX (*P* < .001).

**Conclusion:**

Resistance training and *Tribulus terrestris* administration alone appear to have antiapoptotic effects; however, resistance training combined with Tribulus terrestris administration, especially at higher doses, have more desirable effects than resistance training or *Tribulus terrestris* administration alone on the apoptosis markers.

## Introduction

Nowadays, the use of energetic substances has become a complex problem in sport, so that the role of anabolic-androgenic steroids (AASs) is undeniable.^1^ AASs are synthetic derivatives of the male sex hormone (testosterone) that play an important role in body growth.^2^ One of the problems among athletes, especially among bodybuilding athletes, has been the increasing prevalence of the habit of using steroid drugs.^3^ Abuse of AASs has been reported to lead to overapoptosis of the heart tissue.^2^ Studies in rats show that the use of supraphysiological doses of anabolic steroid drugs causes pathological myocardial hypertrophy and increases apoptosis in isolated ventricular myocytes. It has been reported that 28 days of 5 mg/kg daily administration of stanozolol significantly increased preapoptotic and apoptotic neurons in the cornu ammonis (CA)1, CA2, CA3, and dentate gyrus areas of the hippocampus in rats.^4^ In response to apoptotic stimuli, a variety of internal and external signals regulate the expression of genes that control the onset of apoptosis. In the internal pathway, genes express proteins such as BAX that initiate apoptosis and activate apoptotic inhibitor proteins such as BCL-2.^5^ From the physiological point of view of the heart, apoptosis in this tissue has been introduced as an important way in monitoring various types of nonpathological cell events and occurs from 2 internal pathways (dependent on members of the BCL-2 and BAX family) and the external pathway (or death receptors).^2^ In other words, the release of apoptotic agents from mitochondria to cytosol activates procaspase 9, then caspase 9, and finally, caspase 3 activation as the executive caspase as well as the common chapter of all internal and external apoptotic pathways. Although many studies have been conducted on the effect of different intensities of exercise on skeletal and cardiac muscle cell apoptosis, there are still ambiguities in this regard;^6^,^7^ however, it has been reported that exercise can modulate the apoptotic process of cardiac myocytes.^6^ Resistance training (RT), included in most sports as an important component of a training program, plays a role in the rehabilitation and prevention of injury. RT plays an important role in improving athletic performance through increasing muscle strength, power, speed, hypertrophy, muscle endurance, motor function, balance, and coordination.^7^ RTs due to multiple stages of activity and recovery may increase oxidative stress and have different effects on different body systems as well as apoptotic processes.

In addition to exercise activities, it has been reported that antioxidant foods can have antiapoptotic effects. Studies show that *Tribulus terrestris* plant contains steroids, saponins, flavonoids, alkaloids, unsaturated fatty acids, vitamins, tannins, resins, potassium nitrate, aspartic acid, and glutamic acid.^8^ In this regard, there is a great deal of research evidence on *T. terrestris* plant and its beneficial effects on various diseases.^8^ Effective substances of *T. terrestris* in saponins have been reported to have a protective effect against left ventricular myocardial infarction in an experimental model of hyperlipidemia in rats.^9^ In addition, the administration of *T. terrestris* reduces blood glucose levels and peroxidase indices as well as restores decreased levels of glutathione peroxidase and superoxide dismutase and increased levels of malondialdehyde to the normal levels.^10^ In addition, the antiapoptotic properties of *T. terrestris*-derived saponins have been demonstrated in the cerebral cortex neurons of rats.^8^ Moreover, Wang et al.^11^ reported that *T. terrestris*-derived saponins reduce cellular damage and thereby attenuate cell apoptosis by activating the protein kinase C signaling pathway.^11^

Given the widespread use of stanozolol by athletes and their adverse effects on the heart tissue and the broad and unregulated administration of these drugs to athletes and youth by disqualified individuals and also given the lack of observational study investigating the simultaneous effect of *T. terrestris* and RT, this study aimed to investigate the antiapoptotic effects of RT along with *T. terrestris* administration in the heart tissue of rats exposed to stanozolol.

## Materials and Methods

In this experimental study, 35 Sprague Dawley male rats weighing 150–200 g with an average age of 8 weeks were purchased. After a week of adaptation to the laboratory environment (humidity of 45–55%, dark-light cycle of 12–12 hours, and temperature of 23±2 °C), the rats were divided into 7 groups containing 5 rats each, including (1) the sham (Sh) group (treated with normal saline), (2) the stanozolol-treated group, (3) stanozolol+50 mg/kg *T. terrestris* (ST50)-treated group, (4) stanozolol+100 mg/kg *T. terrestris* (ST100)-treated group, (5) stanozolol+RT (SRT)-treated group, (6) stanozolol+RT+50 mg/kg *T. terrestris* (SRTT50)-treated group, and (7) stanozolol+RT+100 mg/kg *T. terrestris* (SRTT100)-treated group. During 8 weeks, groups 2–7 received 5 mg/kg stanozolol peritoneally per day^12^; groups 5–7 performed RT for 3 sessions per week^13^; and groups 3, 4, 6, and 7 received daily certain doses of *T. terrestris* peritoneally.^14^ This study was approved by the Animal Experiment Ethics Committee of Marvdasht Branch of Islamic Azad University (Code: IR.IAU.M.REC.1399.004). All experiments involving the animals were conducted according to National Institutes of Health Guide for the Care and Use of Laboratory Animals.

A statistical power analysis was performed by G*Power software (version 3.1.9.7.Germany) (BAX: effect size = 5.21, α = 0.05, critical F = 2.44, and power = 0.99; BCL-2: effect size = 6.29, α = 0.05, critical F = 2.44, and power = 0.99; P53: effect size = 4.58, α = 0.05, critical F =2.44, and power = 0.99; caspase 3: effect size = 10.03, α = 0.05, critical F = 2.44, and power = 0.99; BAX/BCL-2: effect size = 4.65, α = 0.05, critical F = 2.44, and power = 0.99).

It should be noted that all rats had free access to food (standard food pellets, including crude protein [23%], crude fat [3.5–4.5%], crude fiber [4–4.5%], ash maximum [10%], calcium [0.95–1%], phosphorus [0.65–0.75%], salt [5–5.5%], humidity maximum [10%], lysine [1.15%], methionine [0.33%], methionine+cysteine [0.63%], threonine [0.72%], and tryptophan [0.25%]) and water.

At 48 hours after the last RT session and stanozolol and *T. terrestris* administration, the rats were anesthetized with ketamine 10% (50 mg/kg) and xylazine 2% (10 mg/kg), and the heart tissue was isolated by laboratory experts and then frozen in liquid nitrogen and stored at −70 °C.

For molecular analysis at the gene expression level, RNA was extracted from the heart tissue using FavorPrep Tissue Total RNA Mini Kit (FavorPrep, Kaohsiung, Taiwan), and then the purity and concentration of RNA were evaluated by optical density measurements on a NanoDrop Lite Spectrophotometer (Thermo Fisher Scientific, Waltham, MA, USA). After extraction of high purity and high concentration RNA from all studied samples, cDNA synthesis was performed by RevertAid First Strand cDNA Synthesis kit (Thermo Fisher Scientific). Reverse transcriptase-quantitative polymerase chain reaction (qPCR) was performed using the ABI Biosystems StepOne and the RealQ Plus 2x Master Mix Green (Ampliqon, Odense, Denmark). The *B2m* housekeeping gene was also used as an internal control of the qPCRs. The qPCR conditions were set for 10 minutes at 94 °C, followed by 40 cycles of 15 seconds at 94 °C, 60 seconds at 60 °C, and the final melt curve stage for product specificity analysis. The amplification signals of different samples were normalized to the B2m cycle threshold, and then the livak method (2^−ΔΔCt^) was applied for comparing the mRNA levels of the different groups, which are represented as fold change in data analysis. The primer sequences are reported in Table 1.

### Resistance Training Protocol

Rats performed RT using a 1-meter high ladder, 4-cm stair distance, and 85° slope, with RT starting with 30% of body weight in the first week and ending with 100% of the rats’ body weights in the eighth week. It should be noted that to warm up, the rats initially climbed the training staircase for 4 repetitions without weight. In addition, each training session consisted of 4 sets (first set with 50%, second set with 75%, third set with 90%, and fourth set with 100% of the bodyweight set for that week) and 2 repetitions (climbing the stairs twice). The interval between each set was 2–3 minutes, and the interval between each repetition was 40–60 seconds.^13^

### *Tribulus Terrestris* Preparation

To prepare *T. terrestris* extract, the fruit of this plant was first milled, and then 100 g of the powder was immersed in 80 ml of 70% alcohol. This solution was then kept in the laboratory for 3 days. After 3 days, the solution was first passed through a paper filter, and the liquid part was purified by a vacuum machine, and the dry extract of the plant was obtained. Subsequently, after the concentration of the extract by normal saline, rats received daily doses of 50 and 100 mg/kg peritoneally.^14^

### Statistical Analysis

To investigate the normality of data, the Kolmogorov-Smirnov test was used, and to analyze the data, 1-way analysis of variance with Tukey’s posthoc tests was used in Statistical Package for the Social Sciences software, version 21, (IBM SPSS Corp.; Armonk, NY, USA) (*P* ≤ .05).

## Results

**Figure 1:**
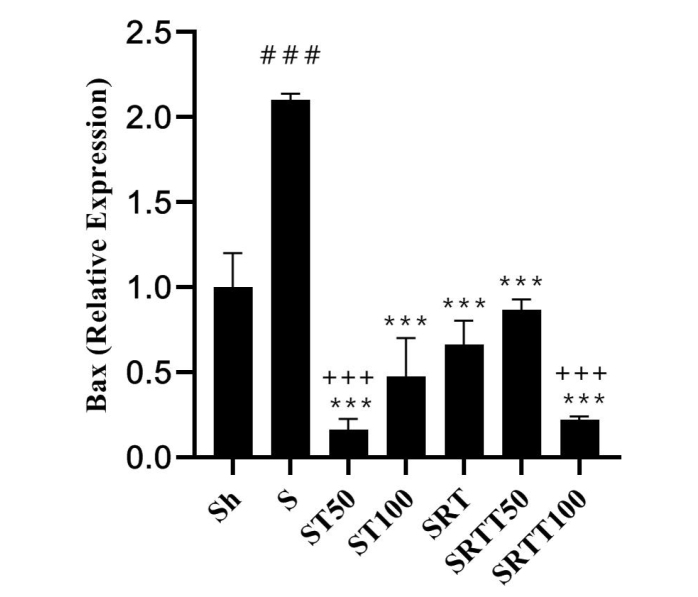
*Bax* gene expression levels in the 7 research groups.

**Figure 2:**
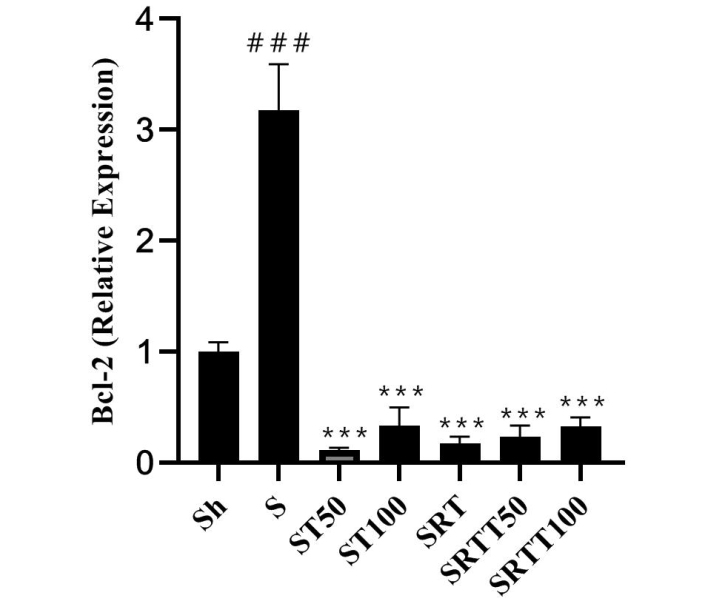
*Bcl-2* gene expression levels in the 7 research groups.

**Figure 3:**
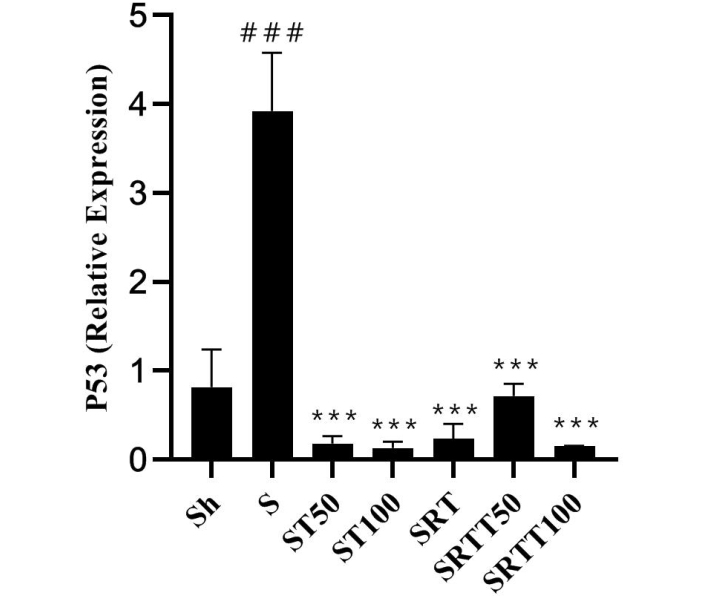
*P53* gene expression levels in the 7 research groups.

**Figure 4:**
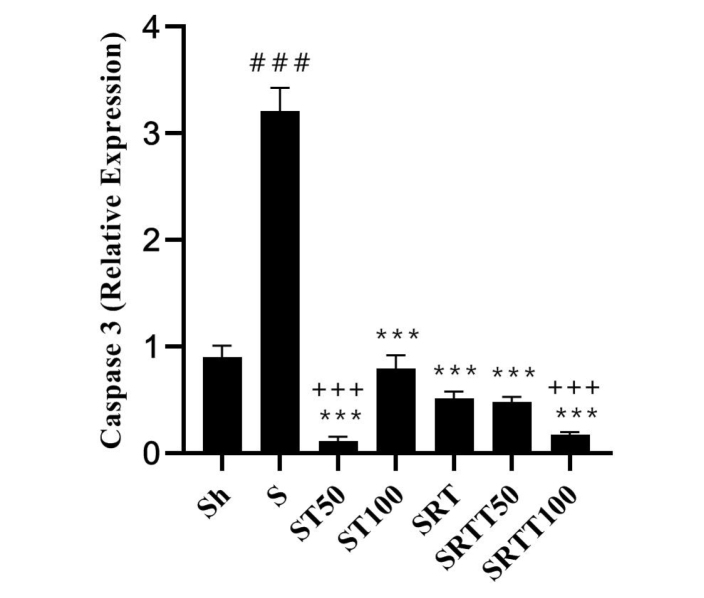
*Caspase 3* gene expression levels in the 7 research groups.

**Figure 5:**
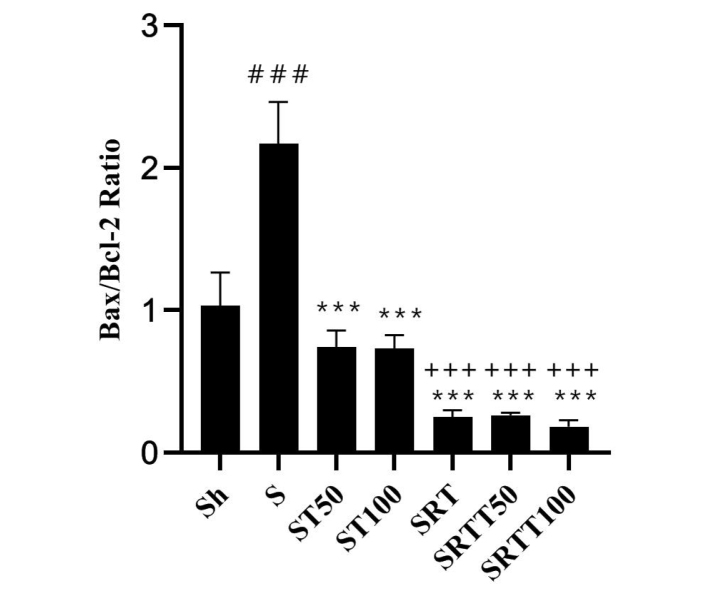
*Bax-to-Bcl-2* ratio in the 7 research groups.

Gene expression levels of *Bax*, *Bcl-2*, *P53*, *caspase 3*, and *Bax*/*Bcl-2* ratio are reported in Figures 1–5, respectively. The results showed that BAX levels in the stanozolol group were significantly higher than those in the Sh group (*P* = .001); however, BAX levels were significantly lower in the ST100, ST50, SRT, SRTT100, and SRTT50 groups than in the stanozolol group (*P* = .001). In addition, the levels were significantly lower in SRTT100 and ST50 groups than in SRT and SRTT50 groups (*P* = .001) (Figure 1).

BCL-2 levels were significantly higher in the stanozolol group than in the Sh group (*P* = .001); however, their levels were significantly lower in the ST100, ST50, SRT, SRTT100, and SRTT50 groups than in the stanozolol group (*P* = .001) (Figure 2).

P53 levels were significantly higher in the stanozolol group than in the Sh group (*P* = .001); however, P53 levels were significantly lower in the ST100, ST50, SRT, SRTT100, and SRTT50 groups than in the stanozolol group (*P* = .001) (Figure 3).

Caspase 3 levels were significantly higher in the stanozolol group than in the Sh group (*P* = .001); however, the levels were significantly lower in the ST100, ST50, SRT, SRTT100, and SRTT50 groups than in the stanozolol group (*P* = .001). In addition, the levels were significantly lower in the SRTT50 and SRTT100 groups than in the ST100, SRT, and SRTT50 groups (*P* = .001) (Figure 4).

BAX/BCL-2 ratio in the stanozolol group was significantly higher than in the Sh group (*P* = .01); however, the ratio was significantly lower in the ST100, ST50, SRT, SRTT100, and SRTT50 groups than in the stanozolol group (*P* = .001). In addition, the ratio was significantly lower in the SRT, SRTT100, and SRTT50 groups than the ST100 and ST50 groups (*P* = .001) (Figure 5).

## Discussion

The results of this study showed that stanozolol administration significantly increased BAX, BCL-2, P53, and caspase 3 levels and BAX/BCL-2 ratio in the heart tissue of rats; nevertheless, RT, *T. terrestris* administration, and RT in combination with *T. terrestris* administration significantly reduced BAX, BCL-2, P53, and caspase 3 levels and BAX/BCL-2 ratio in the heart tissue of rats exposed to stanozolol, such that RT combined with 100 mg/kg *T. terrestris* administration had a more favorable effect than RT combined with 50 mg/kg *T. terrestris* administration on enhancing the level of BAX in the heart tissue of rats exposed to stanozolol. Anabolic steroid abuse has always been associated with cardiovascular disease, and the severity of these diseases depends on the individual differences of the consumers.^15^ Stanozolol activates calcium ion-dependent voltage-gated channels, potassium ion-dependent voltage-gated channels, and calcium ion-activated potassium channels by inhibiting nitric oxide.^16^ Disrupting intracellular calcium ion and its excessive releasing from sarcoplasmic reticulum leads to activation of caspase 3, which itself is involved in activating the inducers of apoptosis and cytochrome C release in cardiac myocytes.^17^

Consistent with the findings of this study, administration of 5 mg/kg stanozolol for 6 weeks induced bradycardia in rats, whereas in rats that received stanozolol and performed swimming training, only hypertension, ventricular hypertrophy, and increased ventricular weight relative to total heart weight was observed.^12^ Stanozolol administration also reduced stretching and development of myocardial muscle fibers and was associated with impaired systolic and diastolic volume, but stanozolol administration in trained rats improved cardiac tissue function. Calcium modulation after severe cardiac contractions at ensuing exercise appears to be a possible mechanism of it.^18^ In another study, nandrolone decanoate increased blood pressure, heart-to-body weight ratio, cardiac sympathetic activity, testosterone, caspase 3, and pathologic changes in the heart tissue of rats. However, training along with nandrolone had no significant effect on the heart rate, heart-to-body weight, and blood pressure in the control group.^19^ Nonetheless, given the lower levels of BCL-2 in the group that received training and stanozolol than in the control group, the BAX/BCL-2 ratio seems to be a more sensitive and accurate index for measuring cell susceptibility to apoptosis.^20^ Confirming the findings of this study, the expression levels of P53 and caspase 3 were also reduced.

The results also showed that *T. terrestris* administration significantly reduced the levels of BAX, BCL-2, P53, and caspase 3 and BAX/BCL-2 ratio in the heart tissue of rats exposed to stanozolol; in addition, these effects were not dose dependent, and doses of 50 and 100 mg/kg had the same effects on decreasing apoptosis. Research suggests that *T. terrestris* consumption has androgenic effects; therefore, it can be used as a natural androgenic to increase free sex hormones in the blood.^21^ In addition, by activating mitogen-activated protein kinase, *T. terrestris* leads to increased expression of lipid metabolism proteins and subsequently results in decreased levels of oxidative stress and inflammatory factors such as NF-κB. In addition, increased IL-10 and the inhibition of IL-1β, TNF-α, IL-6, and IL-8 as well as anti-inflammatory and antiapoptotic effects of *T. terrestris* have been reported in studies.^22^ Studies show that duration and dose are 2 important factors in the effectiveness of this medicinal plant. In this vein, the antioxidant effect of *T. terrestris* extract in ischemia-induced cardiac tissue was dose dependent, such that a dose of 100 mg/kg *T. terrestris* was reported to be more favorable than doses of 1 and 10 mg/kg *T. terrestris*.^23^ In addition, daily consumption of 250 mg/kg *T. terrestris* for 8 weeks had a significant effect on the antioxidant increase and decrease in oxidative stress in the heart tissue of rats with myocardial infarction^24^; 100, 200, and 400 mg/kg *T. terrestris* consumption improved the serum levels of amylase and myeloperoxidase in the pancreatic tissue of rats with pancreatic injury as well.^25^

The results of this study showed that RT combined with *T. terrestris* administration significantly reduced BAX, BCL-2, P53, and caspase 3 levels and BAX/BCL-2 ratio in the heart tissue of rats exposed to stanozolol, although RT combined with higher doses of *T. terrestris* caused a more favorable decrease of BAX. Studies have shown that the modulation of calcium after cardiac contractions,^18^ the reductions of oxidative stress and caspase 3, and increase in VEGF in the heart tissue of rats could partly inhibit the apoptotic effects of stanozolol.^19^ In addition, through the mechanism of activation of mitogen-activated protein kinase, *T. terrestris* consumption leads to an increase in the expression of lipid metabolism proteins, which has lipid-lowering effects in the blood. Furthermore, it decreases lipid levels, oxidative stress, and inflammatory factors such as NF-κB; increases IL-10; inhibits IL-1β, TNF-α, IL-6, and IL-8; and thus induces both anti-inflammatory and antiapoptotic effects.^22^ Accordingly, these 2 interventions appear to have synergistically different pathways that have potentiated each other’s effects in reducing the apoptosis of cardiac tissue under stanozolol intoxication. Therefore, more favorable results at higher doses of *T. terrestris* combined with RT can support the hypothesis of this study. On the other hand, no study was found to have investigated the antiapoptotic effects of *T. terrestris* consumption on the heart tissue poisoned by anabolic steroid abuse, and hence, this study was limited in making a comparison with similar studies. However, some studies have investigated the concurrent effects of *T. terrestris* consumption and exercise training; for example, 1,250 mg/day *T. terrestris* consumption for 4 weeks reduced creatine kinase and muscle injury caused by high-intensity training.^26^ In addition, 120 mg/kg *T. terrestris* consumption for 8 weeks improved the athletic performance of rats that were obese, and also, the levels of IGF-1 and beta-adrenergic-1 receptors were significantly higher in the group that received training and *T. terrestris* consumption.^27^ Owing to the decreased apoptosis in the heart tissue of rats after RT and *T. terrestris* consumption, it seems that this study is limited by the different methods of reporting cardiac function such as electrocardiography, tunnel apoptosis assessment, and measurement of oxidative stress levels. It is therefore suggested that in future studies, these factors should be reported along with other cardiac tissue apoptosis factors. In addition, the inability to measure the protein levels of apoptosis markers by enzyme-linked immunosorbent assay (ELISA) and western blotting methods is another limitations to this study. Therefore, it is also suggested that to confirm the findings of this study in future studies, the effect of different intensities of RT combined with *T. terrestris* administration on the protein levels of apoptosis markers (measurement by ELISA and western blotting methods) should be investigated.

In conclusion, it appears that 8 weeks of RT, 50 mg/kg *T. terrestris* administration, and 100 mg/kg *T. terrestris* administration alone can enhance the apoptosis markers in heart tissue of rats exposed to stanozolol; however, RT combined with *T. terrestris* administration, especially at higher doses (100 mg/kg), have more desirable effects on *Bax* gene expression than any single intervention in the heart tissue of rats exposed to stanozolol. Therefore, it should be considered that by enhancing apoptosis, RT with *T. terrestris* extract administration has protective effects on cardiac tissue in case of intoxication with stanozolol. Accordingly, these 2 interventions can be a good way to reduce cardiac impairments in the case of intoxication with stanozolol.

**Main Points**

- Stanozolol can increases levels of Bax, Bcl-2, P53, caspase 3 and Bax/Bcl-2 ratio.
- Resistance training, 50 and 100 mg/kg Tribulus terrestris and resistance training along with Tribulus terrestris can decrease the Bax, Bcl-2, P53, caspase 3 and Bax/Bcl-2 ratio in rats exposed to S.
- Resistance training along with 100 mg/kg Tribulus terrestris has more effect than SRTT50 on decreased levels of Bax in rats exposed to S.
- Resistance training and Tribulus terrestris administration alone appear to have anti-apoptotic effects, however RT simultaneously with T administration, especially at higher doses, have more desirable effects than RT and T alone on the apoptosis markers in the heart tissue of rats exposed to S.

## Figures and Tables

**Table 1:** The Sequence of Primers Used in the Study

Gene	Primer sequence	Size (bp)
B2m	Forward: 5′- CGTGCTTGCCATTCAGAAA -3′ Reverse: 5′-ATATACATCGGTCTCGGTGG -3′	244
Bax	Forward: 5′- CTGCAGAGGATGATTGCTGA -3′ Reverse: 5′- GATCAGCTCGGGCACTTTAG -3′	174
Bcl-2	Forward: 5′- ATCGCTCTGTGGATGACTGAGTAC-3′ Forward: 5′- AGAGACAGCCAGGAGAAATCAAAC -3′	134
P53	Reverse: 5′- GGCTCCGACTATACCACTATCC -3′ Reverse: 5′- GAGTCTTCCAGCGTGATGATG -3′	104
Caspase- 3	Forward: 5′- AGCTTGGAACGCGAAGAA -3′ Reverse: 5′- GCTTCCATGGATAGTCTTTGTTTC-3′	98

bp: base pair.
